# Inhalation of titanium dioxide nanoparticles during gestation alters placental bioenergetics in a sex-related manner in rats

**DOI:** 10.1016/j.placenta.2025.06.018

**Published:** 2025-06-27

**Authors:** Talia Seymore, Changjiang Guo, Alyssa Bellomo, Julia Herbert, Debra Laskin, Andrew Gow, Phoebe Stapleton

**Affiliations:** a Department of Pharmacology and Toxicology, Ernest Mario School of Pharmacy, USA; b Environmental and Occupational Health Sciences Institute, Rutgers University, Piscataway, NJ, USA

**Keywords:** Placental metabolism, Placental bioenergetics, Fetal growth restriction, Particulate matter, Tissue slices, Nanoparticles

## Abstract

**Introduction:**

Epidemiological and experimental studies support an association between exposure to particulate matter during pregnancy and the development of fetal growth restriction (FGR). The etiology of FGR is often attributed to poor nutrient delivery. Glucose is the primary energy substrate for fetal growth and an important energy source for placental tissue function; therefore, the health of the feto-placental unit depends on sufficient delivery of this nutrient to the tissue. Preeclampsia and FGR are associated with altered placental metabolism; moreover, the underlying causes and progression of these pathologies are influenced by fetal sex. The goal of this study was to investigate sex-related metabolic changes in the placenta after gestational exposure to particulate matter.

**Methods:**

Sprague Dawley rats were exposed to nano-titanium dioxide (nano-TiO_2_) aerosols throughout pregnancy [gestational day (GD) 6-GD20]. For these studies, we developed a novel precision-cut placenta slice model for analysis of tissue bioenergetics using an Agilent Seahorse Analyzer.

**Results:**

Exposure of pregnant rats to nano-TiO_2_ aerosols (9.74 ± 0.11 mg/m^3^) resulted in an overall decrease in placental metabolic function with an increased reliance on glycolytic ATP production. Reductions in maximum metabolic function were sex-related, revealing that female placentas are more sensitive to environmentally induced metabolic changes.

**Discussion:**

These data show that there are sex-related mechanisms within the glycolytic pathway for increased glucose utilization. As increased metabolism of glucose by the placenta can reduce fetal glucose delivery, it may contribute to adverse effects on fetal growth induced by nano-TiO_2_.

## 1. Introduction

Adverse fetal development is a growing concern particularly in environments with a high burden of air pollutants, including particulate matter [1-5]. Particulate matter encompasses a heterogenous mixture of particles of varying sizes and sources, including tobacco smoke, diesel exhaust, gasoline combustion, and roadway dust [6-9]. Notably, these studies support an association between exposure to particulate matter and fetal growth restriction (FGR), a developmental condition defined by the small size of neonates relative to their gestational age [10]. FGR has been linked to the development of metabolic, cardiovascular, and respiratory diseases in adulthood [11,12]. It has been suggested that the developmental origins of these disorders are correlated with reprogramming of placenta nutrient handling, and subsequently the fetus, as it adapts to survival within a hostile gestational environment [13-15]. Commonly referred to as the Barker hypothesis [11], this provides rationale to investigate mechanisms underlying the development of FGR with the goal of developing novel interventions.

The placenta is a vital organ that develops during pregnancy to facilitate the exchange of nutrients from the maternal circulation and metabolic waste from the fetal compartment [16]. Fetal growth is largely driven by the transport of nutrients from the maternal blood to and across the placenta [17]. Glucose is the primary energy substrate for fetal growth and fetal access is dependent on its transport across the placental barrier [18]. For example, of the glucose that reaches the sheep placenta, only 20 % is transported into the fetal compartment, with the majority (50 %) metabolized within the placenta or converted to lactate (30 %) [19,20]. In humans, glucose utilization is closer to 50:50, with half of the glucose that reaches the placenta being transported to the fetus and the other half metabolized in the placenta [21]. This indicates that under homeostatic conditions, there is a delicate balance between glucose transport and glucose metabolism that ensures proficient placental function and fetal nutrient delivery. Importantly, there is evidence to support an association between particulate matter exposure and altered nutrient transport/uptake of the placenta, but these studies did not assess functional metabolic, or bioenergetic capacity after exposure [22-24].

The placenta primarily generates ATP through aerobic respiration and most of this energy is utilized for protein synthesis and active transport systems [25]. About 70 % of the oxygen consumed by the placenta is used for oxidative phosphorylation of glucose, resulting in ATP generation by mitochondria [26]. Glucose that enters the placenta is processed through the glycolytic pathway generating ATP and pyruvate [27]. Under aerobic conditions, pyruvate can diffuse into the mitochondria where it enters the tricarboxylic acid (TCA) cycle, generating NADH and FADH_2_. These reducing equivalents enter the electron transport chain leading to the production of 32 ATPs per molecule of glucose [27,28]. Alternatively, under anaerobic conditions, pyruvate is converted into lactate which is then reused in gluconeo-genesis. The development of pregnancy-related diseases like preeclampsia and gestational diabetes has been linked to altered placental metabolism [29,30].

Evidence suggests that the placental response to stress, including maternal disease and air pollution, can be sex-related, including the capacity to modify glucose uptake and mitochondrial respiration [31,32]. In this context, female placentas have a greater adaptive capacity when compared to male placentas [33]. These adaptations are protective mechanisms to ensure the survival of the fetus. Conversely, male placentas capitalize on available nutrients to prioritize fetal growth over the health and survival of the feto-placental unit [34-36]. Based on these findings, we speculate that male placentas may be more susceptible to environmental stressors.

The purpose of this study was to assess metabolic adaptations within the placenta after gestational exposure to xenobiotic ultrafine particles represented by nano-titanium dioxide (nano-TiO_2_). We hypothesize that under these conditions the placenta increases glucose consumption and metabolism, reducing fetal glucose bioavailability, and perpetuating FGR. For these studies, we developed a novel model using precision-cut placenta slices to include all relevant cell types in the labyrinth zone of the placenta and aerosolized engineered nano-TiO_2_ to recapitulate the inhalation of nanoparticles as previously described [37,38]. Although engineered nano-TiO_2_ does not simulate the inherent heterogeneity of ambient air particulate matter, here we have determined the particle size-specific effects of the inhalation of nanoparticles. The biological importance of this size fraction (referred to as ultrafine particulate matter in the ambient air) relates to its ability to translocate across biological barriers like those found in the lung, brain, and placenta [37,39,40]. Overall, nano-TiO_2_ has a low-reactivity as compared to other engineered nanoparticles, with unique properties based on the surface chemistry and crystalline properties of the material [41]. We found that although exposure of pregnant dams to nano-TiO_2_ resulted in reduced placental metabolism, there was an increase in the percentage of glycolysis-derived ATP, accompanied by mitochondrial dysfunction. Moreover, these results were sex-related, with females having a more significant reduction in maximum metabolic activity compared to males. These novel findings are important as they suggest that inhalation of nanoparticles may alter placenta glucose metabolism, resulting in altered nutrient availability for the fetus.

## 2. Methods

### 2.1. Animals and exposure

**Fig. 1.**
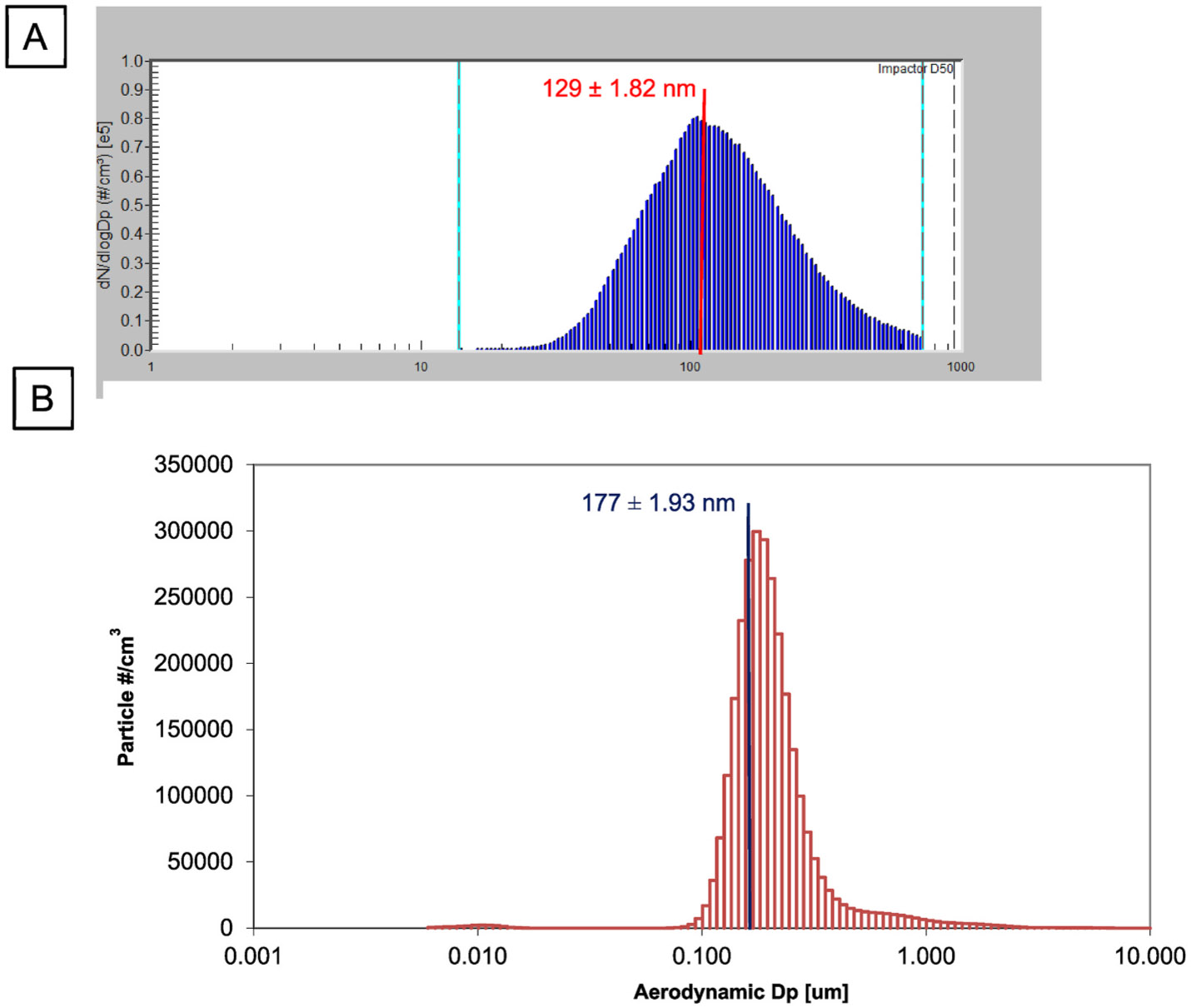
Real-time particle size characterization using electrical mobility (A; SMPS) and aerodynamic diameter (B; ELPI). Data are geometric mean ± geometric SD.

All procedures were approved by the Institutional Animal Care and Use Committee of Rutgers University. Pregnant Sprague Dawley rats were purchased from Charles River Laboratories (Kingston, NY) and delivered on gestational day (GD) 5 to an AAALAC accredited vivarium; food and water were provided *ad libitum*. After 24 h acclimatization, rats were exposed (n = 8) to 9.74 ± 0.11 mg/m^3^ aerosolized nano-TiO_2_ powder (Aeroxide TiO_2_, Parsippany, NJ) for 4–5 h/d, 5 d/wk from GD 6 to GD 19 using a custom designed 84 L whole-body rodent inhalation exposure chamber (IEStechno, Morgantown, WV) and compared to naïve control dams (n = 8) who did not enter the facility. An exposure concentration near 10 mg/m^3^ was used to represent maximum permissible exposure limits for occupational airborne chemical contaminants (Title 8, Article 107) [42]. Particle pulmonary total deposition and deposition after clearance have been previously reported [43]. Dams were euthanized on GD 20 and placental tissues isolated and prepared for metabolic assessment or stored at −80 °C. Bulk nano-TiO_2_ powder was aerosolized by an acoustic drum and delivered to the exposure chamber as previously described [15,43-47]. Temperature, humidity, particle size distribution, and aerosol concentration were monitored in real-time during the exposure. Particle size was measured by aerodynamic diameter using a Scanning Mobility Particle Sizer (SMPS, TSI, Shoreview, MN), which captured particles 1-1000 nm in diameter. Average particle size was calculated as 129 ± 1.82 nm (Fig. 1A). A high resolution electrical low-pressure impactor (HR-ELPI, Dekati, Finland) was used to measure particle size in a wider range (6 nm - 10 μm) using electrical mobility and optical counting, indicating a peak mode particle size of 177 ± 1.93 nm (Fig. 1B). Collectively, these instruments confirm and characterize the nano-TiO_2_ particles within the ultrafine range.

### 2.2. Tissue collection

Dams were weighed (n = 8), anesthetized with isoflurane (5 % induction, 3 % maintenance), and euthanized by pneumothorax and heart removal. The right uterine horn was isolated, excised, and placed in cold (4 °C) physiological salt solution (PSS: 119 mM NaCl, 4.7 mM KCl, 1.17 mM MgSO_4_ 7H_2_O, 1.6 mM, CaCl_2_ 2 H_2_O, 1.18 mM NaH_2_PO4, 24 mM NaHCO_3_, 5.5 mM glucose, and 0.03 mM EGTA) under a dissection microscope. The horn was opened, feto-placental units selected from the center of the horn, and the sex of the attached fetus determined by anogenital distance and confirmed via gonad visualization. The umbilical artery of selected units was carefully identified and separated from the umbilical vein. Placentas were removed from the uterine lining horn, with the umbilical artery attached, and placed in a microvessel chamber (Living Systems Instrumentation, Fairfax, VT) filled with cold PSS. Left uterine horns were isolated, excised, and placed in cold PSS. Remaining fetal sex, fetal weights and placental weights from both horns were recorded and placental efficiency (i.e., fetal weight divided by placental weight) was calculated. 2 male and 2 female placentas from each dam were quartered and snap frozen in liquid nitrogen and stored at −80 °C until RNA isolation.

### 2.3. Preparation of precision-cut placenta slices

The umbilical artery was cannulated with a 4-inch 25-gauge needle and PSS (5–8 mL) was perfused through the artery and placenta to remove red blood cells. Placentas were then perfused with 1 % low melting point agarose (Millipore Sigma, A9414) in Dulbecco’s Modified Eagle Medium (DMEM) (Thermo Fisher Scientific, 21063029) to maintain placental architecture. Placentas were positioned flat on a weight boat with the fetal facing side down. Two samples were collected via punch biopsy (8 mm, Labviva, LV06383547) midway between the metrial gland and the edge of the placenta. It was important to avoid the center after the metrial gland tissue was deemed too fibrous during procedure optimization. Biopsy punches were placed in slicer-derived tissue embedding units, encased with 2 % low melting point agarose and allowed to congeal for 10 min at 4 °C to provide tissue support during tissue slicing. After solidifying, placenta-agarose units were placed into an Alabama R&D Tissue Slicer and 200 μm slices generated and collected in sterile 1x phosphate buffered saline (PBS). Each placenta yielded 20-24 slices. Precision-cut placenta slices were cultured in 24-well plates containing phenol red-free DMEM supplemented with 1 % fetal bovine serum (Thermo Fisher Scientific, 10082147), 1 % penicillin-streptomycin (Millipore Sigma, P4333), and 0.1 % gentamicin (Thermo Fisher Scientific, 15750060). Plates were placed on an orbital shaker in a 37 °C incubator with 20 % O_2_/5 % CO_2_. Culture media was changed 1 h and 3 h after slicing to remove cellular debris. Slices were then left to incubate over night for 18 h to allow the tissue to acclimate to media conditions before preparation for viability assessments and metabolic analyses.

### 2.4. Analysis of precision-cut placenta slice viability

Mitochondrial dehydrogenase function was assessed using a water-soluble tetrazolium salt-1 (WST-1) assay (Cell Proliferation Reagent WST-1 solution kit, Roche). Six slices/placenta were incubated with WST-1 reagent in culture media (1:20 dilution) at 37 °C for 45 min. Mitochondrial dehydrogenase activity was measured spectrophotometrically at 490 nm on a Molecular Devices Spectramax M3 microplate reader. Cell membrane integrity was assessed using a lactate dehydrogenase release assay (LDH, Cytotoxicity Detection Kit Plus, Roche). Triton-X (1:20 dilution) treated slices were used as a positive control. Absorbance was measured in supernatants at 490 nm. All viability data was normalized to total protein and data from the 6 slices were averaged to represent a single placenta. Total protein was determined by soni-cating each slice in 130 μL RIPA lysis buffer (Thermo Fisher Scientific, AAJ63306). Protein content in supernatants was then measured using the Bradford Protein Assay method (Bio-Rad, 5000006).

### 2.5. Analysis of placental bioenergetics

A Seahorse XF96 Analyzer (Agilent Technologies, Santa Clara, CA) was used to measure extracellular acidification rate (ECAR) and oxygen consumption rate (OCR) using the Glycolysis Stress Test, Cell Mitochondrial Stress Test and Real-Time ATP Rate [48-50]. One male and one female placenta from each dam was assessed (n = 8 dams). Punch cuts (~3 mm; Integra^™^) from 5 tissue slices from each placenta were used for each stress test. 3 mm punch cuts were placed in a Poly-D-Lysine coated microplate (Thermo Fischer Scientific) containing ECAR (Agilent Seahorse XF Base Media, pH 7.4 supplemented with 2 mM glutamine) or OCR (Agilent Seahorse XF Base Media, pH 7.4 supplemented with 10 mM glucose, 2 mM glutamine, and 1 mM pyruvate) media for 1 h at 37 °C in a CO_2_-free incubator.

ECAR was measured at baseline and after the sequential addition of 10 mM glucose, 2 μM oligomycin, and 50 mM 2-deoxyglucose (2-DG) for the glycolysis stress test. OCR was also measured at baseline before and after the sequential addition of 2 μM oligomycin, 1 μM carbonyl cyanide-p-trifluoromethoxy-phenylhydrazone (FCCP), and 4 μM rotenone and antimycin A (R/A) for the mitochondrial stress test; and the sequential addition of 2 μM oligomycin, 4 μM R/A, and OCR media for the real-time ATP rate assay. The sequence and mechanistic function of each substrate/inhibitor is as described by the manufacturer [48-50]. Computed endpoints include: spare respiratory capacity [maximal respiration (OCR post FCCP) - basal respiration (initial OCR)], glycolytic capacity (ECAR post oligomycin – ECAR in the absence of glucose), glycolytic reserve (glycolytic capacity – ECAR in response to glucose), non-glycolytic acidification (ECAR in the absence of glucose), proton leak (OCR post oligomycin – OCR post R/A), basal OCR (OCR in the absence of oligomycin), maximal respiration (OCR post FCCP injection – OCR post R/A), non-mitochondrial oxygen consumption (OCR post R/A), coupling efficiency [(ATP production rate)/(basal respiration) x 100], glycolytic ATP production (proton efflux rate), and mitochondrial ATP production (maximal OCR – OCR post oligomycin). Data were analyzed using Agilent Wave Software v. 2.6.3.5. All measurements were normalized to total protein using the Bradford Protein Assay.

### 2.6. RNA extraction and RT-qPCR of mitochondrial DNA (mtDNA)

Placental RNA was extracted from two male and two female placentas per dam (n = 6–9 dams) using TRIzol Reagent (Invitrogen^™^, 15596018) and chloroform. RNA integrity was evaluated using a NanoDrop and confirmed by visualization of 28S and 18S rRNA bands on denaturing gels. Complimentary DNA (cDNA) was generated from RNA (13.2 μg) using Applied Biosystems^™^ High-Capacity cDNA Reverse Transcription Kit and Power SYBR^™^ Green PCR Master Mix was used to measure amplified signals of mtDNA (NCBI Reference Sequence: NC_001665.2). Amplification cycling was conducted using a Quant-Studio Applied Biosystems ViiA7 qPCR machine and mtDNA CT values were normalized to *B-actin* (NCBI Reference Sequence: NM_031144.3). Fold changes were calculated relative to the average of female naïve samples. Primer sequences are listed below.

mtDNA forward: 5′-CGA CGC AGA CAA AAT CCC AT −3′
mtDNA reverse: 5′- GTT TGT TGG GAA TGG AGC GT −3′
*B-actin* forward: 5′- ATG TAC CCA GGC ATT GCT GA −3′
*B-actin* reverse: 5′- AGG GTG TAA AAC GCA GCT CA −3′

### 2.7. Statistical analyses

Statistical analyses were performed, and graphs generated using GraphPad Prism v9 (La, Jolla, CA). Litter characteristics and metabolic endpoints are presented as a dam/litter average and are interrogated with sex as a biological variable by two-way ANOVA analyses with Tukey’s multiple comparisons test. Single variable comparisons between naïve and exposed groups were analyzed using unpaired t-tests and viability assays were analyzed using one-way ANOVA. Repeated measures ANOVA was performed for ECAR and OCR graphs. Data are presented as mean ± SEM and considered significant at p ≤ 0.05. Outliers were removed and identified as values that fell beyond two standard deviations of the mean.

## 3. Results

### 3.1. Litter characteristics

Litter characteristics were recorded for the left and right horns and included maternal weight, litter size, fetal weight, placental weight, and placental efficiency (Table 1). A significant decrease in fetal weight was observed after exposure of animals (n = 23–26 litters) to nano-TiO_2_ (4.21 ± 0.08 g) when compared to unexposed controls (4.39 ± 0.04 g) groups. A subset of these animals (n = 6–8 litters) was used for placental metabolic measurements and assessment of other litter characteristics; fetal growth restriction was not identified in this smaller cohort (Table 1). No differences in maternal weight or litter size were observed between the control and exposed groups. However, exposure caused an overall significant decrease (9 %) in placental weight and a 12 % increase in placental efficiency relative to placenta from naïve animals.

### 3.2. Tissue viability

**Fig. 2.**
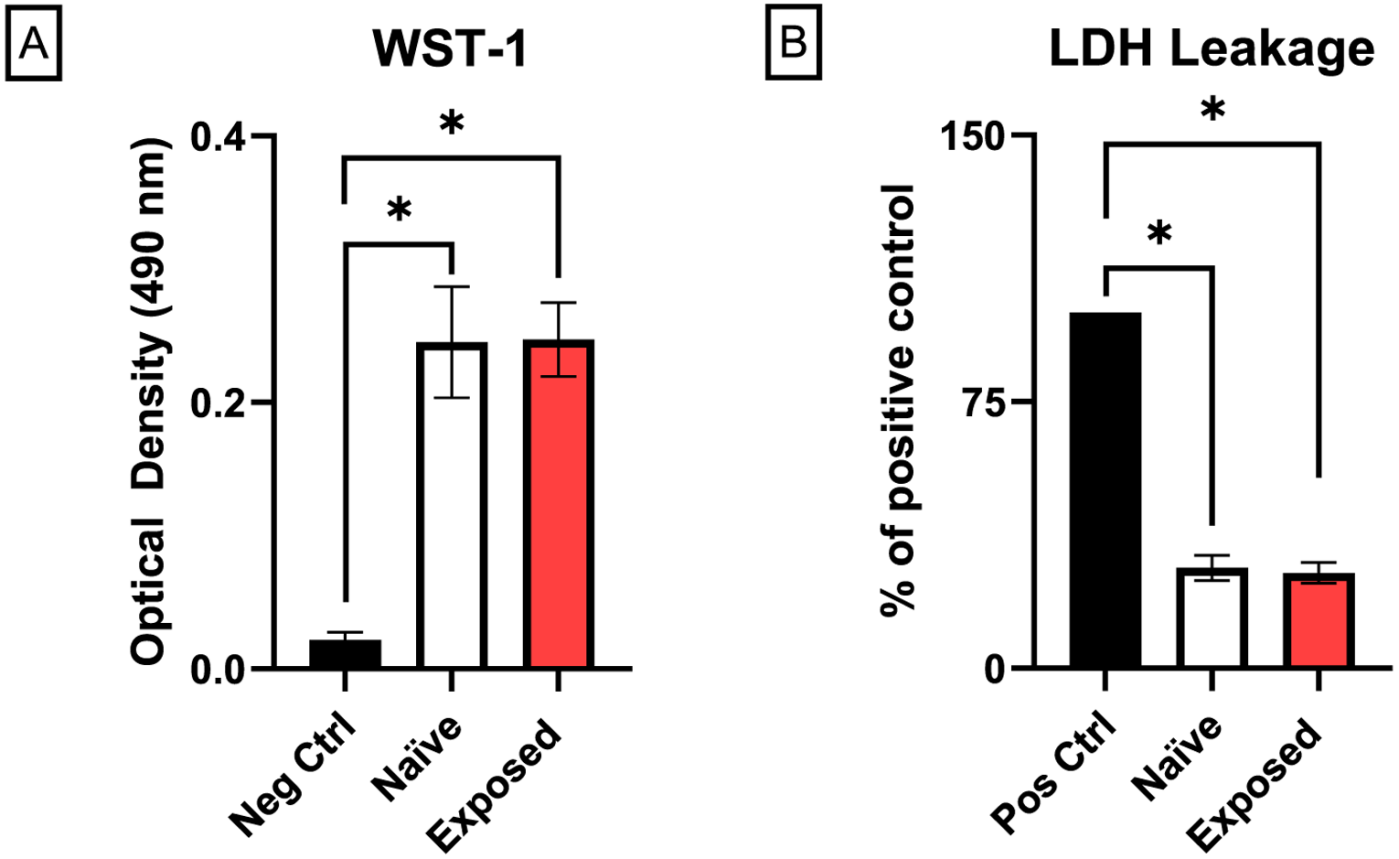
Effects of tissue slicing on cell viability. Mitochondrial dehydrogenase function and cell membrane integrity of placenta slices from naïve and exposed dams were assessed by WST-1 assay (A) and LDH release (B), respectively. Slices treated with Triton-X were used as negative and positive controls for WST and LDH leakage, respectively. Data were normalized to protein concentration. Data are mean ± SEM, n = 6–7 litters/treatment group. *Significantly different (p ≤ 0.05) between indicated groups as determined by one-way ANOVA.

Mitochondrial dehydrogenase function of precision-cut placenta slices from naïve dams and those treated with nano- TiO_2_ was significantly higher than negative control slices, indicating the presence of viable mitochondria (Fig. 2A). LDH leakage from slices of naïve dams and those treated with nano-TiO_2_ was significantly lower than that of positive controls, indicating intact cell membranes (Fig. 2B). Collectively these data confirm the cellular viability of precision-cut placenta slices for bioenergetic analyses.

### 3.3. Measurement of placental bioenergetics

**Fig. 3.**
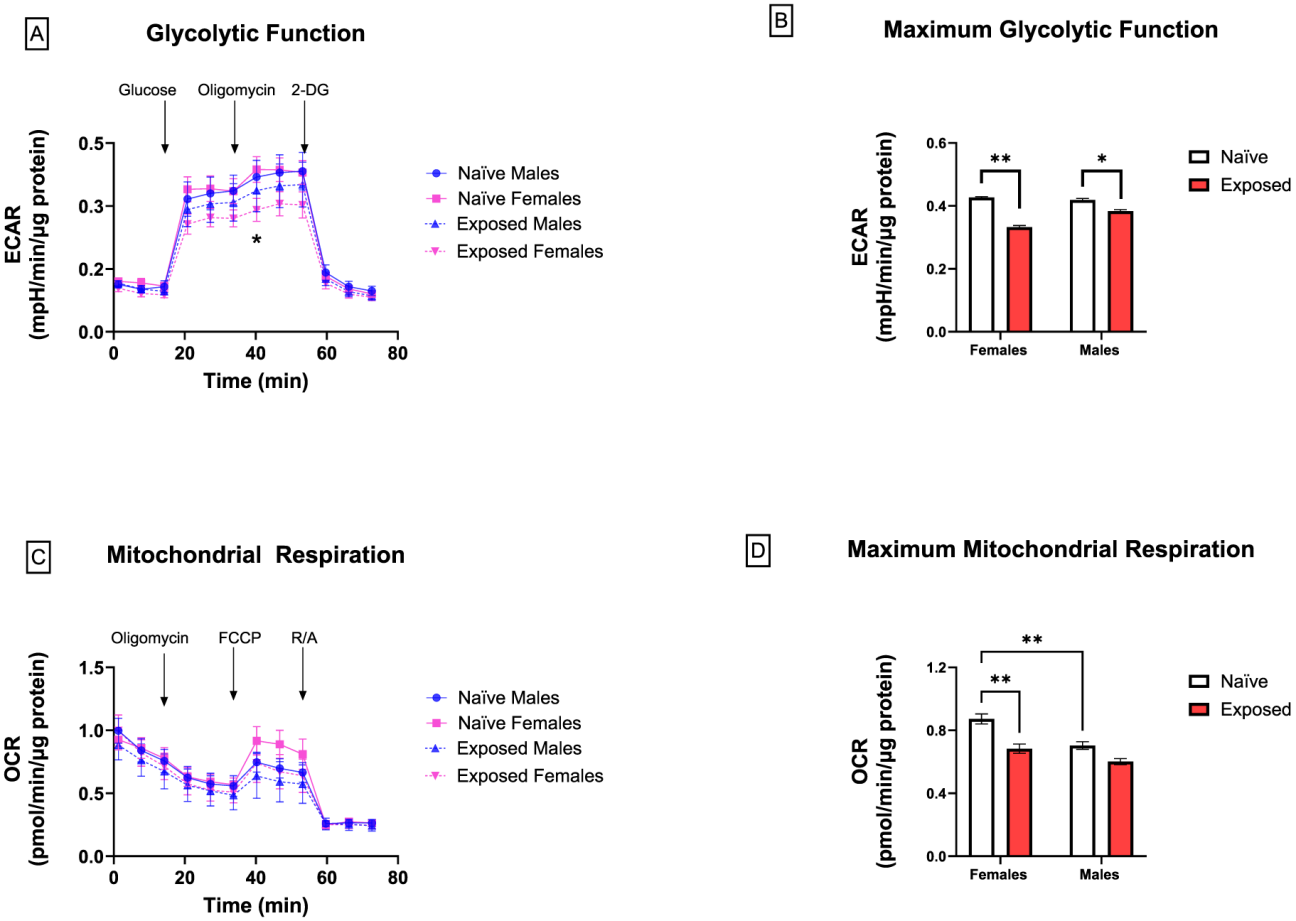
Effects of nano-TiO_2_ inhalation on glycolytic function (A and B) and mitochondrial respiration (C and D). 24 hr after tissue collection, OCR and ECAR of placenta slices from naïve and exposed dams were measured at baseline and after the sequential treatment of the following chemicals given these specific assays: glycolysis stress test [10 mM glucose, 2 μM oligomycin, and 50 mM 2-deoxyglucose (2-DG)] and mitochondrial stress test [2 μM oligomycin, 1 μM carbonyl cyanide-p-trifluoromethoxy-phenylhydrazone (FCCP)] (Significantly different *p ≤ 0.05 between female treatment groups as determined by unpaired *t*-test). Maximum glycolytic and mitochondrial function was assessed by averaging responses to oligomycin and FCCP, respectively (Significantly different *p ≤ 0.05; **p ≤ 0.001 as determined by two-way ANOVA). Mean ± SEM, n = 6–8 litters/treatment group.

**Fig. 4.**
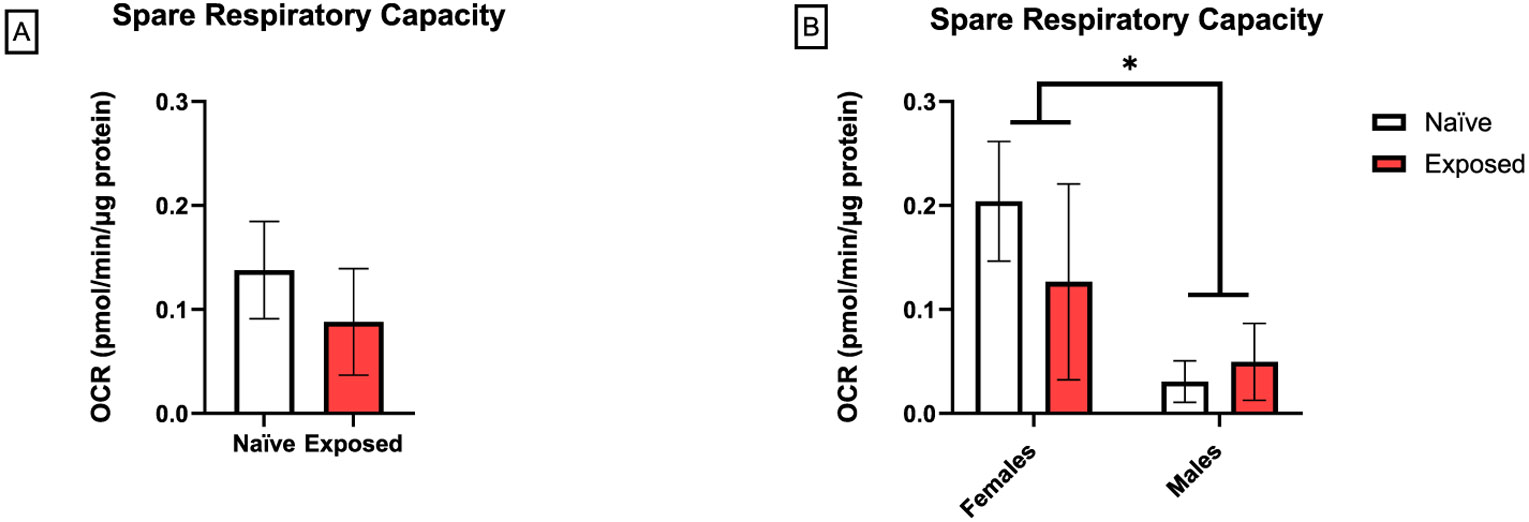
Effect of nano-TiO_2_ inhalation on spare respiratory capacity. Spare respiratory capacity was calculated from the mitochondrial stress test by subtracting basal respiration from maximal respiration (A). Data were stratified by sex to determine sex-related effects (B). Mean ± SEM, n = 6–8 litters/treatment group. *Significantly different (p ≤ 0.05) between indicated groups as determined by two-way ANOVA.

Initially, we assessed the effects of nano-TiO_2_ on glycolytic activity of placental slices as measured by ECAR after the sequential addition of glucose, oligomycin, and 2-DG. Exposed female placentas had a significantly lower ECAR in response to oligomycin stimulation compared to naïve (Fig. 3A). Further evaluation of maximum glycolytic function was calculated by averaging responses to oligomycin stimulation. There was a significant effect of exposure and sex on maximum glycolytic function with a more pronounced effect in females compared to males (20 % vs 8 % reduction, respectively). Moreover, glycolytic function after exposure was significantly lower in female placentas than in males (Fig. 3B). We next measured mitochondrial respiration by analyzing OCR after the sequential addition of oligomycin, FCCP, and R/A. There were no significant differences at individual time points (Fig. 3C). However, further evaluation of maximum mitochondrial respiration was calculated by averaging responses to FCCP stimulation. At baseline, male placentas had a lower maximum mitochondrial function compared to female placentas. Furthermore, there was a significant effect of exposure on reduced maximum mitochondrial respiration. Additional analyses with Tukey’s multiple comparisons test revealed that this response was primarily driven by female placentas (Fig. 3D). However, male placentas exhibited a significantly lower spare respiratory capacity compared to females, regardless of exposure (Fig. 4A and B). There was no effect on glycolytic capacity, glycolytic reserve, non-glycolytic acidification, proton leak, basal OCR, maximal respiration, non-mitochondrial oxygen consumption, or coupling efficiency (data not shown).

### 3.4. ATP production and mitochondrial quantification

**Fig. 5.**
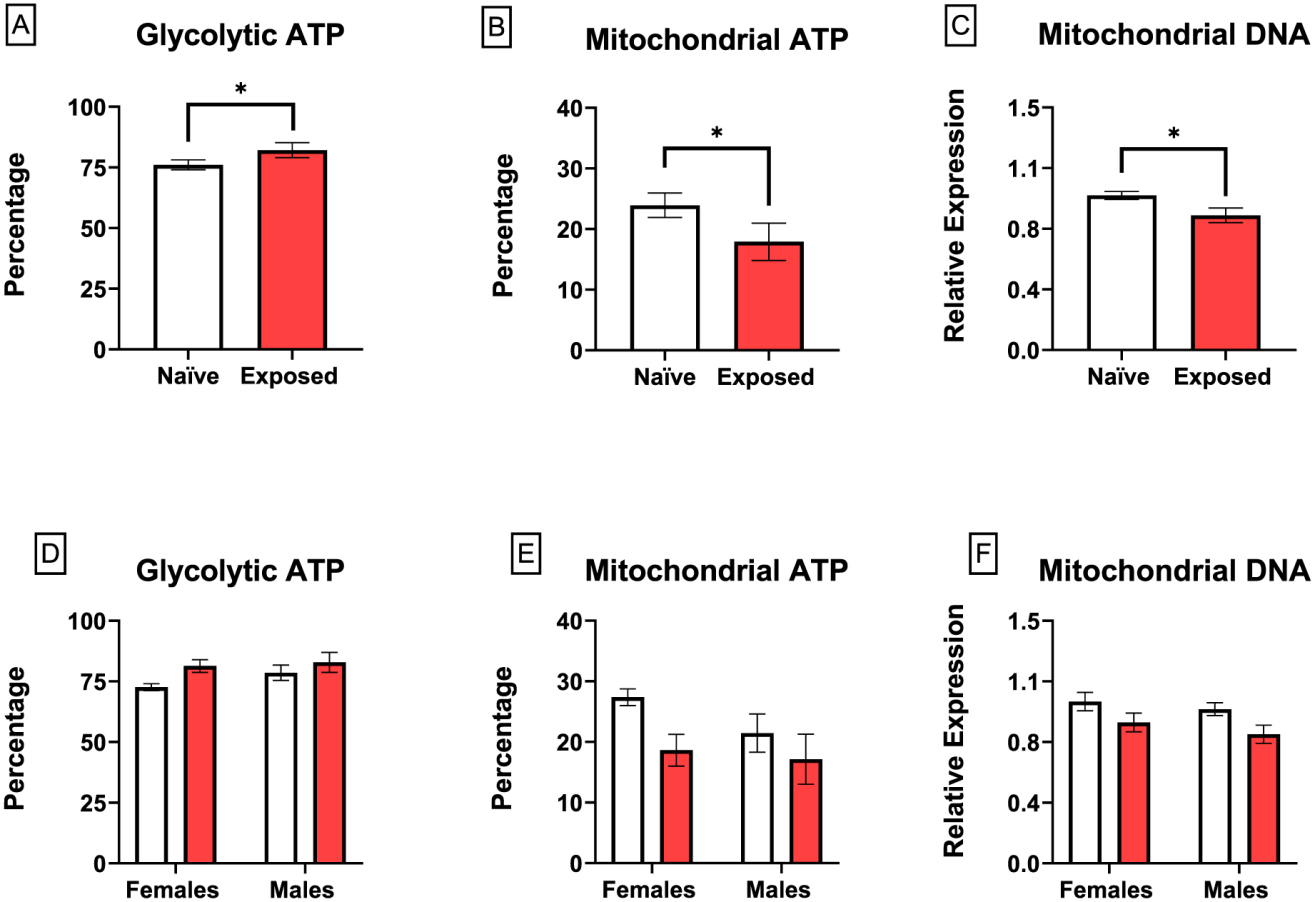
Effect of nano-TiO_2_ inhalation on ATP generation. ATP production was measured at baseline and after the sequential treatment of 2 μM oligomycin, 4 μM R/A, and OCR using a real-time ATP rate assay. Percentage of cellular ATP from glycolysis and oxidative phosphorylation was calculated (A and B). To quantify mitochondria number, mtDNA was measured using RT-qPCR (C). Data were stratified by sex to determine sex-related effects (D, E, and F). Mean ± SEM, n = 6–8 litters/treatment group. *Significantly different (p ≤ 0.05) between indicated groups as determined by two-way ANOVA.

To assess the relative contribution of glycolysis and mitochondrial respiration to total ATP production, we used a real-time ATP rate assay. Glycolytic ATP (Fig. 5A) production was significantly increased in placenta slices from dams exposed to nano-TiO_2_ with a corresponding decrease in mitochondrial ATP production (Fig. 5B). This was associated with a reduction in mitochondrial copy number (Fig. 5C). There were no sex-related effects on glycolytic ATP, mitochondrial ATP, or mitochondrial copy number (Fig. 5D, E, and F). Total ATP production was unaffected between treatment groups (data not shown).

## 4. Discussion

This investigation demonstrated that the labyrinth zone of the placenta exhibits an altered metabolic response after maternal exposure to nano-TiO_2_ during pregnancy. This was characterized by an overall decrease in maximum metabolic function, mitochondrial dysfunction and an increase in glycolytic ATP production. Furthermore, the placental responses are sex-related. While placentas exhibit increased reliance on glycolytic function as a response to nano-TiO_2_ exposure, female placentas have a more pronounced decrease in overall metabolic function compared to male placentas. These findings support our hypothesis that FGR after exposure to nano-TiO_2_ aerosols during gestation may be attributed to reduced glucose bioavailability for passage to the fetus due to increased glucose utilization in the placenta.

Herein, we developed and optimized an innovative approach using an Agilent Seahorse Analyzer to assess and calculate cellular bioenergetic responses to maternal environmental stressors on placenta tissues. This methodology permitted us to assess metabolism within the placental labyrinth zone, a highly heterogenous tissue that allows for the nutrient and waste exchange between the maternal and fetal compartments. The Agilent Seahorse has been used previously to investigate placental bioenergetics in isolated cytotrophoblasts or syncytiotrophoblasts [51-54]. Our results cannot be directly compared to these studies since individual placental cell types are known to display distinct metabolic capacities [55]. Only one other study using whole placental explants from mice included all placental cell types [51]; however, ECAR responses were found to be lower than ours; this was likely due to a low ratio of reactive surface cells relative to the total tissue weight. Other techniques include the use of respirometry to evaluate mitochondrial function using whole tissue or isolated mitochondria, which neglects glycolytic output, cellular acidification, and ATP production [56-59]. In conclusion, the Agilent Seahorse Analyzer offers the most detailed evaluation to assess mitochondrial bioenergetics.

The placenta relies largely on glycolysis for energy production [55,60], a response exacerbated by nano-TiO_2_ exposure during gestation. Known as the Warburg effect, pyruvate is converted to lactate for metabolism in lieu of being shuttled to the mitochondria and incorporated into the TCA cycle [61]. Using our model, we observed that more than 50 % of ATP produced in the placenta is glycolytic, which indicates that the placenta derives most of its energy from glycolytic ATP production; this allows oxygen to be conserved for fetal delivery. While glycolysis generates less ATP than oxidative phosphorylation, the process of ATP generation is significantly faster. As the placenta is a hypoxic organ and the need to provide oxygen to the fetus for growth and development is greater, this compensated metabolism may be beneficial for fetal survival [17]. This metabolic shift in the placenta has also been observed as a response to hypoxia in the uterine environment, a phenomenon known as the Pasteur effect [62-64]. Although we did not evaluate oxygen tension in these studies, reduced uterine perfusion during gestational exposure to nano-TiO_2_ has been documented [15].

In these experiments, we identified placental mitochondrial dysfunction as an outcome of gestational exposure to nano-TiO_2_. These findings are in line with reports of decreases in basal respiration, maximal respiration, and spare capacity in isolated cardiomyocytes from fetal, neonate, and adult offspring after exposure to nano-TiO_2_ during pregnancy [65]. These findings suggest that mitochondrial dysfunction is a shared mechanism of toxicity induced by particulate matter and nano-TiO_2_ [66-69]. Others have also identified disruptions to placental metabolism, including nutrient uptake and metabolic homeostasis, after particulate matter exposure during gestation [23,24,70]. Our previous findings identified translocation of nano-TiO_2_ to the placental and fetal environment after gestational exposure [37,71]; however, it remains to be determined if particle translocation has a direct role in mitochondrial dysfunction.

Examination of maximum metabolic capacity revealed that female placentas are more sensitive to nano-TiO_2_ exposure compared to male placentas. The stark reduction in ECAR of female placentas can suggests lactate retention or a reduction in lactic acid production, indicative of a metabolic shift toward the TCA cycle or the pentose phosphate pathway (PPP) [28]. However, we did not identify an increase in mitochondrial ATP production. This suggests that the PPP pathway is likely responding. Similar observations were described in human placentas compromised by FGR, more specifically, through reductions in phosphofructokinase activity, the rate limiting step of glycolysis, and reductions in lactate production [72]. The PPP involves the breakdown of glucose-6-phosphate to produce NADPH, a process that is enhanced during antioxidant responses [73]. Although oxidative stress was not investigated in this study, it is one of the primary forms of cytotoxicity elicited by nanoparticles and ultrafine particulate matter [74,75]. Female placentas also exhibited a pronounced decrease in maximum mitochondrial respiration, a response that can be protective against mitochondria-derived oxidative stress [76]. Overall, female placentas may be more efficient at shifting their metabolic activity to limit oxidative production and combat oxidative stress-induced cytotoxicity after gestational exposure to nano-TiO_2_ aerosols.

While this study provides insight into the impact of maternal exposure on fetal development, certain limitations should be considered when interpreting the results. Future investigations with environmentally relevant particulate matter should be assessed to verify these findings, as nano-TiO_2_ does not have the diverse physiochemical and characterization properties that are present in ambient particulate matter. These differences (including, but not limited to: crystallinity, shape, chemical composition and purity, chemical mixtures, particle size, endotoxin, functional groups, surface charge) can affect biological responses; therefore, this point should be considered when extrapolating the data. Studies also show that there are differences in metabolic activity between cell populations in the placenta. For example, cytotrophoblast cells have been shown to be more glycolytic and metabolically active than syncytiotrophoblasts cells [55]. Therefore, cytotrophoblast differentiation or changes in the ratio of cell populations would offer insight into the mechanisms behind these observations. In addition to cell population, physiological conditions such as blood flow and shear stress can affect placental bioenergetics [77]. For this reason, to improve this model, future investigations should consider the relevance of physiological conditions when using *in vivo/ex vivo* models such as tissue slices. Furthermore, our precision-cut placenta slices were collected only from the labyrinth zone, the primary site of nutrient exchange in the rodent placenta. Analysis of metabolic parameters in the decidua and junctional zones would offer a more comprehensive understanding of placental metabolism. However, this analysis will require careful identification of the ratio of decidual, junctional, and labyrinth zone(s) within each placental slice sample. In addition, studies were conducted using 20 % O_2_. As the placenta is a hypoxic organ, future studies may require an evaluation of the tissue at an oxygen tension of 8 % to mimic a physiologically relevant environment [78]. Future studies may also need to assess placental bioenergetics in a model of gestational hypoxia (<8 %), a state known to also induce FGR [79,80].

In summary, these studies demonstrate that placental glucose metabolism is sensitive to maternal inhalation of nano-TiO_2_ during pregnancy. Moreover, the reliance on glycolytic metabolism in the placenta is exacerbated after particulate exposure, indicating an increase reliance on placental glucose consumption. Our detailed assessment of cellular bioenergetics provides new insights into sex-related metabolic adaptations of the placenta in response to environmental stress. Future studies are ongoing to evaluate placental glucose transport mechanisms and fetal blood glucose to detail their involvement in the development of FGR. These studies may be useful in assessing the value of using glucose-related interventions to prevent the development of FGR in environments with a high burden of particulate matter.

## Figures and Tables

**Table 1:** Effects of nano-TiO_2_ inhalation on litter characteristics. Maternal weight, litter size, average fetal weight, average placental weight, and average placental efficiency was collected on GD 20. Data are mean ± SEM, n = 6–8 litters/treatment group. *^E^Significantly different exposure factor (p ≤ 0.05) as determined by two-way ANOVA.

	Naïve		Exposed	
Maternal Weight (g)	320 ± 6.1		327 ± 8.1	
Litter Size	11 ± 0.5		12 ± 0.6	
	Female	Male	Female	Male
Fetal Weight (g)	4.1 ± 0.1	4.4 ± 0.1	4.2 ± 0.1	4.5 ± 0.2
Placental Weight (g)	0.52 ± 0.02	0.52 ± 0.01	0.46 ± 0.02*^E^	0.49 ± 0.02*^E^
Placental Efficiency	8.1 ± 0.5	8.4 ± 0.3	9.3 ± 0.4*^E^	9.2 ± 0.4*^E^

## Abbreviations:

ECAR: extracellular acidification rate
FCCP: carbonyl cyanide-p-trifluoromethoxy-phenylhydrazone
FGR: fetal growth restriction
mtDNA: mitochondrial DNA
OCR: oxygen consumption rate
PPP: pentose phosphate pathway
R/A: rotenone and antimycin
TCA: tricarboxylic acid
2-DG: 2-deoxyglucose
